# Native Valve Infective Endocarditis with Severe Regurgitation: What Matters Is Heart Failure

**DOI:** 10.3390/jcm13206222

**Published:** 2024-10-18

**Authors:** Adrián Lozano Ibañez, Paloma Pulido, Javier López Díaz, María de Miguel, Gonzalo Cabezón, Andrea Oña, Pablo Zulet, Adrián Jerónimo, Daniel Gómez, Daniel Pinilla-García, Carmen Olmos, Carmen Sáez, Javier B. Pérez-Serrano, Isidre Vilacosta, Itziar Gómez-Salvador, J. Alberto San Román

**Affiliations:** 1 Servicio de Cardiología, Instituto de Ciencias del Corazón (ICICOR), Hospital Clínico Universitario de Valladolid, 47003 Valladolid, Spain; 2 Centro de Investigación Biomédica en Red de Enfermedades Cardiovasculares (CIBERCV), 28029 Madrid, Spain; 3 Instituto de Investigación Sanitaria del Hospital Clínico San Carlos (IdISSC), 28040 Madrid, Spain; 4 Facultad de Ciencias Biomédicas y de la Salud, Universidad Europea de Madrid, 28005 Madrid, Spain; 5 Sección de Enfermedades Infecciosas, Servicio de Medicina Interna, Hospital Universitario de la Princesa, Instituto de Investigación Sanitaria del H.U. Princesa (IIS-IP), 28006 Madrid, Spain; 6 Department of Medicine, Universidad Complutense de Madrid, 28040 Madrid, Spain

**Keywords:** severe regurgitation, infective endocarditis, heart failure, prognosis

## Abstract

**Background/Objectives:** Heart failure worsens the prognosis of patients with infective endocarditis (IE) and is mainly caused by severe valvular regurgitation. The aim of our investigation is to describe the clinical, epidemiological, microbiological, and echocardiographic characteristics of patients with native left-sided infective endocarditis (NLSIE) with severe valvular regurgitation; to describe the prognosis according to the therapeutic approach; and to determine the prognostic factors of in-hospital mortality. **Methods**: We prospectively recruited all episodes of possible or definite NLSIE diagnosed at three tertiary hospitals between 2005 and 2022. Patients were divided into two groups: patients with severe valvular regurgitation at the time of admission or during hospitalization and patients without severe valvular regurgitation. We analyzed up to 85 variables concerning epidemiological, clinical, analytical, microbiological, and echocardiographic data. **Results**: We recovered 874 patients with NLSIE, 564 (65%) of them with severe valvular regurgitation. There were no differences in mortality among patients with and without severe regurgitation (30.2% vs. 26.5%, *p* = 0.223). However, mortality increased when patients with severe regurgitation developed heart failure (33% vs. 11.4%, *p* < 0.001). Independent factors related to heart failure were age (OR 1.02 [1.01–1.034], *p* = 0.001), anemia (OR 1.2 [1.18–3.31], *p* = 0.01), atrial fibrillation (OR 2.3 [1.08–4.89], *p* = 0.03), *S. viridans*-related IE (OR 0.47 [0.3–0.73], *p* = 0.001), and mitroaortic severe regurgitation (OR 2.4 [1.15–5.02], *p* = 0.019). **Conclusions**: Severe valvular regurgitation is very frequent among patients with NLSIE, but it does not worsen the prognosis of patients unless complicated with heart failure.

## 1. Introduction

Heart failure worsens the prognosis of patients with left-sided infective endocarditis (IE) and represents the main surgical indication in these patients [1,2]. In most patients, heart failure results from severe valvular regurgitation, which affects up to 55% of patients with left-sided IE, according to previous studies [3,4]. Hypothetically, the presence of severe valvular regurgitation in patients with native left-sided infective endocarditis (NLSIE) is associated with an increased risk of heart failure and the need for surgery; it could be caused by more aggressive micro-organisms and might be associated with a poorer outcome. However, these hypotheses remain unsettled, as there is no study focused on this specific group of patients. Given its high prevalence, it is important to know whether severe valvular regurgitation entails clinical or prognostic specific issues.

The aim of our investigation was to describe the clinical, epidemiological, microbiological, and echocardiographic characteristics of patients with NLSIE with severe valvular regurgitation, to analyze the patient prognosis according to the therapeutic approach, and to determine the prognostic factors of in-hospital mortality.

## 2. Materials and Methods

### 2.1. Population

We prospectively recruited all episodes of possible or definite IE diagnosed at three tertiary hospitals between 2005 and 2022 according to diagnostic criteria accepted at each period (modified Duke criteria from 2005 to 2014 and ESC 2015 modified diagnostic criteria from 2015 to 2022). The inclusion criterion for the analysis was NLSIE.

All patients underwent transthoracic (TTE) and transesophageal (TEE) echocardiography performed by imaging experts certified by the Spanish Society of Echocardiography, and the degree of regurgitation was determined according to European Guidelines of Echocardiography present in each period of the study. Quantitative and semiquantitative measures such as Proximal Isovelocity Surface Area (PISA), vena contracta width, pressure half-time, and color Doppler 2D and 3D were used whenever possible [5].

Patients were then divided into two groups: patients with severe valvular regurgitation at the time of admission or during hospitalization and patients without severe valvular regurgitation. Patients with severe regurgitation already known before admission were included in the former group.

The main indications for surgery were heart failure, unresponsiveness to medical treatment, septic shock, persistent signs of infection, fungal endocarditis, and recurrent systemic embolism despite adequate antibiotic therapy. The decision to operate on a patient was always made by the IE-Heart Team, composed of cardiologists, heart surgeons, and experts in infectious diseases.

### 2.2. Variables Studied

We analyzed up to 85 variables concerning epidemiological, clinical, analytical, microbiological, and echocardiographic data at the time of admission and during hospitalization, as well as information about treatment and clinical outcomes. The proportion of missing data was <10% in all analyzed variables.

### 2.3. Definition of Terms

Heart failure was diagnosed by a clinical cardiologist according to the ESC guidelines criteria [6]. Acute onset of symptoms was defined by a cut-off of 15 days between the onset of symptoms and hospital admission. Nosocomial IE was defined as IE occurring 72 h or more after admission to the hospital or when the episode was related to a hospitalary procedure performed in the last 8 weeks [1]. Renal failure was defined as serum creatinine levels greater than 2 mg/dL in patients with previously normal renal function and a creatinine level increase >50% in those with chronic renal insufficiency. Chronic anemia was defined as serum hemoglobin levels lower than 13 g/dL in males and 12 g/dL in females for at least a year. Persistent infection was defined as persistent fever and positive blood cultures after 7 days of adequate antibiotic treatment once other potential causes of fever or the presence of perivalvular complications were excluded [6]. Patients were assessed in all cases with TTE and TEE at the time of diagnosis, and severe valvular regurgitation was defined according to the European Guidelines of Echocardiography [5]. Previous disease on the affected valve was defined as any known valvular disease, independently of its severity or condition, prior to admission. The bicuspid aortic valve was included in “congenital valvular disease”, and mitral valve prolapse was involved in “degenerative valvular disease”. Systemic emboli were diagnosed by imaging techniques such as computed tomography and echography. Referred cases were those patients transferred to the tertiary hospital from other healthcare centers where they were initially admitted.

### 2.4. Statistical Analysis

Continuous variables were reported as the mean ± standard deviation or median [interquartile range, IQR] according to variable distribution (normal or not). Student’s *t*-test or Mann–Whitney U tests were used for comparison among groups. The normal distribution of continuous variables was verified with the Kolmogorov–Smirnov test and q-q plot. Categorical variables were reported as absolute values and percentages and compared with a Chi-square test or Fisher’s exact test when expected frequencies were less than 5.

Multivariable models by logistic regression with the maximum likelihood backward stepwise method were adjusted to analyze the prognostic factors of in-hospital mortality and heart failure in patients with severe valvular regurgitation.

All prognostic factors with a *p* value of less than 0.05 in the univariate model were further entered into the multivariate analysis. Age, gender, diabetes mellitus, chronic renal failure, pulmonary hypertension, vegetation, heart failure, septic shock, renal failure, *Streptococcus viridans*, *Staphylococcus aureus*, and surgery were included in the model for in-hospital mortality. Age, degenerative valve disease, anemia, atrial fibrillation (AF), mitroaortic severe regurgitation, *Streptococcus viridans*, *Enterococcus* spp., coagulase-negative staphylococci, and negative cultures were included in the model for heart failure.

For all adjusted models, the ratio variable/event was controlled to avoid overfitting. Odds ratios (ORs), adjusted for each of the variables included, along with their 95% confidence intervals (95% CI), were calculated. Noncollinearity was checked among the variables. The area under the receiver operating characteristic curve (ROC curve) was used to measure the discriminatory capacity. Calibration was evaluated with the Hosmer–Lemeshow test and with plots.

Kaplan–Meier curves were estimated for 1-year mortality and compared between groups with the log-rank test.

Statistical analysis was performed using R software, version 3.6.1 (R Project for Statistical Computing). No imputation of missing data was performed. All tests were two-sided. Differences were statistically significant when the *p* value was <0.05.

## 3. Results

**Figure 1:**
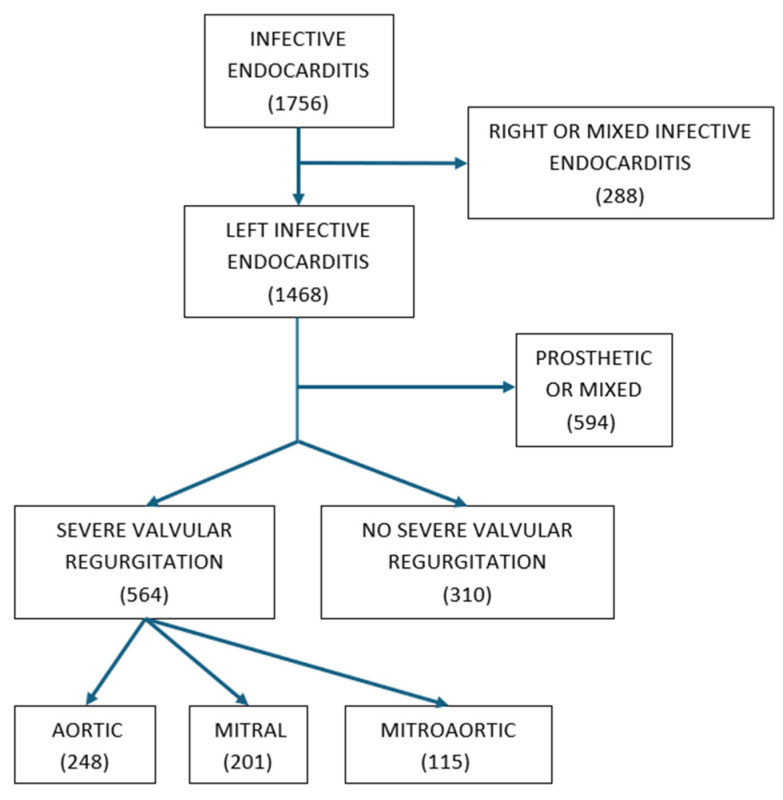
Eligibility criteria and sample size.

Of the 1756 patients with IE included in our database, 1468 were left-sided, and 874 of those were NLSIE. Of these, 564 (65%) had severe valvular regurgitation: 248 had aortic regurgitation (44%), 201 had mitral regurgitation (35%), and 115 had both mitral and aortic severe regurgitation (21%). The remaining 310 patients (35%) had no, mild, or moderate regurgitation. Table 1 shows the characteristics of patients with and without severe regurgitation (Figure 1).

### 3.1. Characteristics of Patients with and without Severe Regurgitation

Table 1 compares the epidemiologic, clinical, microbiologic, echocardiographic, and prognostic characteristics of patients with and without severe valvular regurgitation.

Of note, patients with severe regurgitation were more frequently referred from other hospitals (73% vs. 55%; *p* = 0.001). Previous valvular disease of the infected valve in patients with severe regurgitation was less frequent than in patients without severe regurgitation (46% vs. 54.3%; *p* = 0.027). The most frequent initial manifestation in patients with severe regurgitation was heart failure (44.4% vs. 23.2%; *p* < 0.001), while other manifestations such as septic shock (20% vs. 14.1%; *p* = 0.025) and stroke (21% vs. 14.5%; *p* = 0.014) were more frequent in patients without severe regurgitation.

From a microbiological point of view, positive blood cultures at the time of admission showed no difference (83.3% vs. 83.9%, *p* = 0.825). Interestingly, 19.2% of patients with severe regurgitation had persistent positive blood cultures (after 72 h of admission) versus 30.3% of patients without severe regurgitation (*p* = 0.004). Intriguingly, *S. aureus* was more frequent in patients without severe regurgitation (27.1% vs. 17.2%, *p* = 0.001) and *S. viridans* in patients with severe regurgitation (22% vs. 14.2% *p* = 0.005).

The most common surgical indication among patients with severe regurgitation was heart failure (93.4% vs. 48.1%; *p* < 0.001). Uncontrolled infection and prevention of emboli were more frequent in patients with no severe regurgitation (34.8% vs. 63.2%; *p* < 0.001 and 27.4% vs. 40.6%; *p* = 0.009, respectively). Some patients had more than one surgical indication.

### 3.2. Mortality in Patients with Severe Valvular Regurgitation

**Figure 2:**
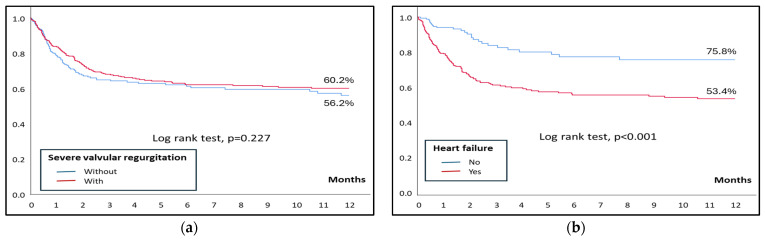
(**a**) Kaplan–Meier curves for 1-year mortality in patients with and without severe regurgitation. (**b**) Kaplan–Meier curves for 1-year mortality in patients with severe regurgitation, with and without heart failure.

Remarkably, in-hospital mortality was similar among patients with and without severe regurgitation (30.2% vs. 26.5%, *p* = 0.223), as well as 1-year mortality (Figure 2). However, patients with severe regurgitation who developed heart failure at any point had a higher in-hospital mortality than those who did not (33% vs. 11.4%; *p* < 0.001), with similar findings in the analysis of 1-year mortality (Figure 2). Table 2 shows the analysis of mortality in patients with severe regurgitation. Independent predictors of mortality in patients with severe regurgitation include heart failure and are shown in Table 3.

### 3.3. Heart Failure in Patients with Severe Regurgitation

Table 4 compares the characteristics of patients with severe regurgitation to those with and without heart failure.

Patients with severe regurgitation who had heart failure were older (*p* < 0.001), had more degenerative valvular disease (28.1% vs. 19.1%, *p* = 0.023), as well as chronic kidney disease (13% vs. 5.6%, *p* = 0.008), anemia (26.8% vs. 12.9%, *p* < 0.001), multiple valve involvement (22.9% vs. 15.1%, *p* = 0.03), and AF (15.5% vs. 5%, *p* < 0.001).

Patients’ microbiological profile showed a lower frequency of infections by *S. viridans* (16.7% vs. 33.5%, *p* < 0.001) and a higher frequency of *Enterococcus* spp. (15.6% vs. 9.5%, *p* = 0.005) and coagulase-negative staphylococci (12.2% vs. 6.7%, *p* = 0.046).

Table 5 shows those factors that were independently associated with heart failure in patients with severe valvular regurgitation. Remarkably, *S. viridans* infection was associated with not developing heart failure (*p* = 0.001).

## 4. Discussion

Scant information is available regarding the impact of severe valvular regurgitation on patients with NLSIE despite it being a common finding among these patients. Our results shed light on the course of these patients, with the largest cohort studied to our knowledge. The main findings were the following: (1) Despite being very frequent, severe valvular regurgitation among patients with NLSIE was not associated with higher in-hospital mortality in our population; (2) Prognosis worsened if patients with severe regurgitation developed heart failure; (3) Predictors of heart failure in patients with NLSIE and severe regurgitation were age, AF, anemia and involvement of both the mitral and aortic valves.

The prevalence of severe regurgitation in our cohort was surprisingly high compared to others [3,4,7], probably due to a selection bias, as all patients with endocarditis and surgical indications from other nearby primary and secondary centers are referred to our hospital. If we exclude all referred patients, the prevalence of severe regurgitation was 55%, resembling other studies’ frequencies. Another baseline difference in our cohort was a higher prevalence of *S. viridans* in the baseline cohort [3,4,7], which showed a significant correlation with severe regurgitation. As described by previous works [8], heart failure was less frequent in patients with *S. viridans*-related IE as well. The subacute onset of endocarditis caused by this micro-organism may explain why severe regurgitation is better tolerated from a clinical standpoint.

On the other hand, nonsevere regurgitation was surprisingly more frequent in *S. aureus*-related IE. Its more aggressive course and association with septic shock and stroke could explain alternative mechanisms for heart failure other than valvular regurgitation, such as renal failure, hyperdynamic circulation, or arrhythmias (as later mentioned).

Heart failure has been largely described as a mortality risk factor in previous studies, with some of them reaching 34% mortality, and has been included in predictive models of in-hospital mortality [3,4,9,10,11]. Recent work focused on patients with IE and surgical indication also proved heart failure to be independently associated not only with all-cause mortality but also with major adverse events, including all-cause death, hospitalizations, and IE relapses [12]. The main finding of our investigation was that in patients with NLSIE and severe regurgitation from our cohort, it was not the dysfunction of the valve but heart failure that was the main feature related to mortality (11% vs. 33%). We identified several variables related to an increased risk of heart failure in this group of patients, which could help clinicians stratify the severity of the disease when severe regurgitation is present. It could also help in deciding when to refer a patient admitted for NLSIE to a center with surgical facilities.

The presence of AF in patients with IE has previously been described as a mortality risk factor and predictor of heart failure [13]. Interestingly, severe regurgitation was not more frequent in patients with AF, suggesting an independent mechanism of heart failure. Furthermore, new-onset AF had a poorer outcome than chronic AF [13]. The loss of atrio-ventricular synchrony, rapid ventricular rate, and the absence of atrial contraction raises filling pressures and pulmonary capillary pressures [14]. Our study shows that AF strongly increases the risk of heart failure and is associated with greater mortality.

As expected, severe regurgitation of multiple valves was also related to a higher risk of heart failure than single valve involvement [15]. Aortic regurgitation (AR) results in an increased end-diastolic volume (EDV), which increases the regurgitant flow of an incompetent mitral valve, thus raising capillary wedge pressure and heart failure symptoms. Furthermore, AR and MR both end up in a decreased forward cardiac output (AR initially increases systolic volume but not organ perfusion), resulting in increased activation of the renin–angiotensin–aldosterone system and worsening heart failure [14]. A previous study already described how bivalvular regurgitation led to more heart failure without increased mortality, which was attributed to a higher surgery rate [16]. Another study, however, found that patients with bivalvular IE had higher mortality, although early surgery increased survival in this set of patients [17]. On the other hand, the severe regurgitation of both mitral and aortic valves in a patient with endocarditis is not always caused directly by an infection of both valves. It has already been described how chronic AR can lead to increased LV dilation, which causes an increased sphericity of LV, changing the position and orientation of papillary muscles and interfering with the coaptation of mitral leaflets, leading to an increased MR [13].

Another important factor to consider is the time dimension, as the hemodynamic behavior of acute regurgitation differs from chronic regurgitation. Dilation and increased compliance of the left ventricle (LV) derived from chronic severe regurgitation allows the accommodation of excess end-diastolic volume and the maintenance of forward systolic volume. On the contrary, the acute setting of severe regurgitation not only limits LV adaptation to increased volume but, in the case of aortic regurgitation, also results in an increased LV end-diastolic pressure exceeding that of the left atrium, which leads to the premature closure of the mitral valve during diastole, limiting LV filling and further decreasing systolic volume. In acute mitral regurgitation, the left atrium does not have time to adapt to increased volume, and capillary wedge pressure increases drastically [18,19]. This particularity in the timing of regurgitation suggests that patients without previous significant valvular disease may have a worse hemodynamic tolerance to acute valvular disease and a poorer outcome. However, since valvular disease is also a risk factor for IE, the correlation between those two variables remains a difficult aspect to address.

Nevertheless, early surgery should be targeted to IE patients who develop heart failure or those at a high risk of doing so, irrespective of the number of valves infected.

From a clinical standpoint, our work enhances the importance of simple and readily available clinical markers that help identify patients with NLSIE and severe regurgitation who have worse prognosis because of the high likelihood of developing heart failure and death.

We are aware of several limitations. Although data were prospectively collected, the study is retrospective, and our conclusions are hypothesis-generating at most. An assessment of regurgitation was not evaluated by an independent core laboratory, but it was undertaken in the local imaging units. However, those are busy units with long-recognized expertise in endocarditis, in which guidelines are tightly followed. The inclusion of patients with severe regurgitation already known at admission in the group of patients with severe regurgitation is questionable, given that the hemodynamic behavior of chronic and acute regurgitation is different [14,20,21,22]. Nonetheless, few patients had known severe regurgitation (*n* = 84), and the results did not change if those patients were excluded from the analysis.

## 5. Conclusions

To summarize, severe valvular regurgitation is very frequent among patients with NLSIE, but it does not worsen the prognosis of patients unless it is complicated with heart failure. We identified several factors associated with the development of heart failure. Future studies should test whether preventive surgery in those patients may improve outcomes.

## Figures and Tables

**Table 1:** Descriptive study of patients with left-sided native valve infective endocarditis (LSNVIE).

	Total (*n* = 874)	Without Severe Valvular Regurgitation (*n* = 310)	With Severe Valvular Regurgitation (*n* = 564)	*p* Value
Epidemiologic variables				
Age	64 ± 15.7	65 ± 15.2	62 ± 15.5	<0.001
Male	68.4% (598)	55.5% (172)	75.5% (426)	<0.001
Referred	48.8% (422)	36.3% (111)	55.7% (311)	<0.001
Nosocomial	19.7% (173)	24.9% (77)	17.1% (96)	0.005
Previous disease on affected valve	45.7% (382)	50.8% (153)	42.9% (229)	0.027
Rheumatic valvular disease	9.2% (80)	15.9% (49)	5.5% (31)	<0.001
Degenerative valvular disease	27.6% (241)	32% (99)	25.2% (142)	0.031
Congenital heart disease	6.9% (60)	3.9% (12)	8.5% (48)	0.01
Previous endocarditis	2.2% (19)	1% (3)	2.8% (16)	0.07
Previous cardiac surgery	2.5% (22)	4.9% (21)	1.2% (13)	0.001
Clinical variables at admission				
Acute onset	47.8% (416)	60.3% (185)	41% (231)	<0.001
Heart failure	36.9% (321)	23.2% (71)	44.4% (250)	<0.001
Septic shock	16.2% (140)	20% (61)	14.1% (79)	0.025
Stroke	14.9% (130)	18.2% (56)	13.1% (74)	0.043
Atrial fibrillation	13% (111)	14.7% (44)	12.1% (67)	0.278
Microbiologic variables				
Positive blood cultures	83.7% (694)	83.3% (245)	83.9% (449)	0.825
*Streptococcus gallolyticus*	6.2% (55)	6.1% (19)	6.4% (36)	0.877
*Streptococcus viridans*	19.2% (168)	14.2% (44)	22% (124)	0.005
*Enterococcus* spp.	12.4% (108)	10% (31)	13.7% (77)	0.114
Other streptococci	9.2% (80)	10.3% (32)	8.5% (48)	0.379
*Staphylococcus aureus*	20.7% (181)	27.1% (84)	17.2% (97)	0.001
Coagulase-negative staphylococci	10% (87)	9% (28)	10.5% (59)	0.494
Gram-negative bacilli	3.3% (29)	4.5% (14)	2.7% (15)	0.144
Fungus	1% (9)	1.9% (6)	0.5% (3)	0.075
HACEK * group	0.7% (6)	0.6% (2)	0.7% (4)	0.999
Anaerobic bacteria	1% (9)	1% (3)	1.1% (6)	0.999
Echocardiographic variables				
Aortic endocarditis	56.3% (492)	41.6% (129)	64.4% (363)	<0.001
Mitral endocarditis	59.8% (523)	66.8% (207)	56% (316)	0.002
Aortic and mitral endocarditis	16.1% (141)	8.4% (26)	20.4% (115)	
Evolutive variables				
Surgery performed	59.7% (522)	36.5% (113)	72.5% (409)	<0.001
Surgery indication: heart failure	80.3% (419)	45.1% (51)	90% (368)	<0.001
Surgery indication: uncontrolled infection	39.1% (204)	59.3% (67)	33.5% (137)	<0.001
Surgery indication: embolic prevention	28.9% (151)	38.1% (43)	26.4% (108)	0.019
Mortality				
In-hospital mortality	27.9% (242)	30.4% (93)	26.5% (149)	0.223

* HACEK: *Haemophilus*, *Aggregatibacter*, *Cardiobacterium*, *Eikenella,* and *Kingella*. Gram-negative bacteria associated with endocarditis.

**Table 2:** Analysis of mortality in patients with severe valvular regurgitation.

	Total (*n* = 564)	In-Hospital Mortality (*n* = 149)	No In-Hospital Mortality (*n* = 415)	*p* Value
Age	62.0 ± 15.5	69.3 ± 12.9	59.4 ± 15.6	<0.001
Left ventricular ejection fraction (%)	61.3 ± 10.1	59.5 ± 11.9	62.0 ± 9.3	0.031
Male	75.5% (426)	68.5% (102)	78.1% (324)	0.019
Referred	55.7% (311)	56.8% (84)	55.4% (227)	0.77
Degenerative valvular disease	25.2% (142)	39.6% (59)	20% (83)	<0.001
Atrial fibrillation	12.1% (67)	17% (25)	10.3% (42)	0.03
*Streptococcus viridans*	22% (124)	10.1% (15)	26.3% (109)	<0.001
*Staphylococcus aureus*	17.2% (97)	32.9% (49)	11.6% (48)	<0.001
Vegetation	91.5% (516)	97.3% (145)	89.4% (371)	0.003
Mitroaortic severe regurgitation	10.3% (58)	14.1% (21)	8.9% (37)	0.074
Ruptured chordae tendineae	17% (96)	18.8% (28)	16.4% (68)	0.503
Heart failure	68.2% (382)	86.3% (126)	61.8% (256)	<0.001
Septic shock	8.4% (47)	18.5% (27)	4.8% (20)	<0.001
Renal failure	25.2% (141)	43.8% (64)	18.6% (77)	<0.001
Cardiac surgery	72.5% (409)	50.3% (75)	80.5% (334)	<0.001

**Table 3:** Independent factors associated with mortality in patients with severe valvular regurgitation.

	Univariate		Multivariate	
	OR (95%CI)	*p* Value	OR (95%CI)	*p* Value
Age	1.051 (1.035–1.067)	<0.001	1.036 (1.035–1.067)	<0.001
Male	0.610 (0.402–0.924)	0.61		
Diabetes mellitus	1.777 (1.157–2.729)	<0.001		
Chronic kidney disease	2.785 (1.612–4.814)	<0.001		
Pulmonary hypertension	2.106 (1.415–3.134)	<0.001		
Vegetation	4.299 (1.517–12.180)	0.006	3.614 (1.163–11.230)	0.026
Heart failure	3.888 (2.331–6.486)	<0.001	2.947 (1.607–5.406)	<0.001
Septic shock	7.829 (4.730–13.293)	<0.001	4.232 (2.268–7.895)	<0.001
Renal failure	5.453 (3.622–8.211)	<0.001	3.190 (1.995–5.101)	<0.001
*Streptococcus viridans*	0.313 (0.176–0.558)	<0.001		
*Staphylococcus aureus*	3.736 (2.370–5.891)	<0.001	2.187 (1.221–3.914)	0.008
Cardiac surgery	0.243 (0.164–0.368)	<0.001	0.354 (0.213–0.588)	<0.001

**Table 4:** Comparison between patients with severe valvular regurgitation who developed heart failure and patients who did not develop heart failure.

	Total (*n* = 564)	No Heart Failure (*n* = 179)	Heart Failure (*n* = 385)	*p* Value
Age (years old)	62.0 ± 15.5	57 ± 16.15	64.2 ± 14.8	<0.001
Left ventricular ejection fraction (%)	61.3 ± 10.1	62.1 ± 9.7	61 ± 10.2	0.234
Degenerative valvular disease	25.2% (142)	19.1% (34)	28.1% (108)	0.023
Diabetes	21.8% (123)	16.3% (29)	24.4% (94)	0.03
Cancer	11.4% (64)	5.6% (10)	14% (54)	0.003
Anemia	22.4% (126)	12.9% (23)	26.8% (103)	<0.001
Atrial fibrillation	12.1% (67)	5% (9)	15.5% (58)	<0.001
*Streptococcus viridans*	22% (124)	33.5% (60)	16.7% (64)	<0.001
*Staphylococcus aureus*	17.2% (124)	14.5% (26)	18.5% (71)	0.246
*Enterococcus* spp.	13.7% (77)	9.5% (17)	15.6% (60)	0.049
Coagulase-negative staphylococci	10.5% (59)	6.7% (12)	12.2% (47)	0.046
Pulmonary hypertension	28.7% (161)	14.1% (25)	35.4% (136)	<0.001
Mitroaortic severe regurgitation	10.3% (58)	5.6% (10)	12.5% (48)	0.012

**Table 5:** Independent predictors of heart failure in patients with severe valvular regurgitation.

	Univariate		Multivariate	
	OR (95%CI)	*p* Value	OR (95%CI)	*p* Value
Age	1.029 (1.017–1.041)	<0.001	1.022 (1.009–1.034)	0.001
Degenerative valvular disease	1.651 (1.069–2.551)	0.024		
Anemia	2.470 (1.509–4.043)	<0.001	1.997 (1.179–3.313)	0.01
Atrial fibrillation	3.456 (1.671–7.146)	0.001	2.303 (1.084–4.893)	0.03
*Enterococcus* spp.	1.765 (1.001–3.122)	0.05		
*Streptococcus viridans*	0.397 (0.263–0.598)	<0.001	0.469 (0.303–0.725)	0.001
Coagulase-negative *staphylococci*	1.941 (1.003–3.757)	0.049		
Negative cultures	2.075 (1.016–4.238)	0.045		
Mitroaortic severe regurgitation	2.407 (1.188–4.876)	0.015	2.405 (1.153–5.017)	0.019

## Data Availability

Data are contained within the article.
